# Analysis of AWS Rekognition and Azure Custom Vision Performance in Parking Sign Recognition

**DOI:** 10.3390/s25195983

**Published:** 2025-09-26

**Authors:** Maria Spichkova, Amanda Severin, Chanakan Amornpatchara, Fiona Le, Thuc Hi Tran, Prathiksha Padmaprasad

**Affiliations:** School of Computing Technologies, RMIT University, Melbourne, VIC 3000, Australia; s3989597@student.rmit.edu.au (A.S.); s4052194@student.rmit.edu.au (C.A.); s3999450@student.rmit.edu.au (F.L.); s3996832@student.rmit.edu.au (T.H.T.); s3973503@student.rmit.edu.au (P.P.)

**Keywords:** parking sign recognition, Computer Vision, AWS, Azure, AI

## Abstract

Automated recognition and analysis of parking signs can greatly enhance the safety and efficiency of both autonomous vehicles and drivers seeking navigational assistance. Our study focused on identifying parking constraints from the parking signs. It offers the following novel contributions: (1) A comparative performance analysis of AWS Rekognition and Azure Custom Vision (CV), two leading services for image recognition and analysis. (2) The first AI-based approach to recognising parking signs typical for Melbourne, Australia, and extracting parking constraint information from them. We utilised 1225 images of the parking signs to evaluate the AI capabilities for analysing these constraints. Both platforms were assessed based on several criteria, including their accuracy in recognising elements of parking signs, sub-signs, and the completeness of the signs. Our experiments demonstrated that both platforms performed effectively and are close to being ready for live application on parking sign analysis. AWS Rekognition demonstrated better results for recognition of parking sign elements and sub-signs (F1 scores of 0.991 and 1.000). It also performed better in the criterion “No text missed”, providing the result of 0.94. Azure CV performed better in the recognition of arrows (F1 score of 0.941). Both approaches demonstrated a similar level of performance for other criteria.

## 1. Introduction

Reading and analysing parking signs manually can be a challenging task, especially while driving. Simple signs with only one or two elements are relatively easy to interpret. However, many parking signs are quite complex. They often present different restrictions for various time periods and may also show different rules for the areas on the right and left of the sign pole. This complexity is particularly problematic in large cities, where parking signs can easily confuse drivers. One potential solution to this issue is to implement automated analysis of parking signs using AI-based image recognition and analysis technology. Then, in this context, we can view vehicles as mobile sensors equipped to detect and relay information about their surroundings, including critical signage that informs driving decisions.

There are a number of prior works on traffic and parking sign recognition; see, for example, [1,2,3,4,5]. This demonstrates the importance of this research direction as well as a practical need to obtain a working solution, which might be a valuable addition for autonomous driving, as well as provide much-needed decision support for drivers preferring to drive manually. Some of the approaches focused on Traffic Sign Recognition (TSR), while other approaches target Parking Sign Recognition (PSR).

In both cases, the approaches aim to cover both the detection of a sign and the recognition of the information presented on the sign. TSR methods [6] typically address the signs that provide traffic-related information, warnings, or instructions to drivers. PSR methods [7] concentrate on the signs that present parking instructions and regulations. While both TSR and PSR require very high accuracy, the environmental and driving constraints for them are generally different. The timing constraints for TSR methods are stricter and require faster responses from the system. For instance, when a car is travelling at 100 km/h and approaches a speed limit sign indicating “60 km/h”, the information about the speed reduction must be processed rapidly to ensure the car can slow down in time. The parking signs should typically be detected at a slower speed when the car is slowing down to look for a potential parking spot. On the other hand, the parking signs contain many elements presenting complex parking constraints.

Our study focuses on AI-based extraction of the parking constraints, i.e., on the PSR methods. The PSR research area is less explored than TSR, but it is currently expanding to provide practical support for drivers. AWS Rekognition and Azure Custom Vision are two leading services for image recognition and analysis. In our previous studies, we analysed the applicability of computer vision technologies, including AWS Rekognition and Google Cloud Vision approaches, for semi-automated meter-reading of non-smart meters [8], where these approaches demonstrated solid results. Therefore, it would make sense to analyse their current capacity for parking sign analysis. Parking signs vary significantly from one country to another. Moreover, in some countries, parking signs differ even in different states and territories, or even between local councils. An overview of the datasets widely used in the TSR field has been introduced in [9]. It presents the following datasets of German traffic signs [10], traffic signs from Belgium and the Netherlands [11], and traffic signs from China. However, to our best knowledge, there is no equivalent database for parking signs, especially for Australia. Therefore, our study is the first to use AI-based approaches for the recognition of parking signs typical in Melbourne, Australia.

Contributions: In our study, we present a comparative performance analysis of AWS Rekognition and Azure Custom Vision performance in identifying parking constraints from the parking signs. Our aim was to answer the following research question: (RQ) How can parking signs be analysed using AWS Rekognition and Azure Custom Vision?

We utilised 1225 images of the parking signs (Melbourne, Australia) to evaluate the AI capabilities for analysing these constraints. Both platforms were evaluated based on their accuracy in recognising parking sign elements and sub-signs, as well as the completeness of the signs. We also assessed their ability to accurately recognise text elements and maintain the correct order of the text. Additionally, we examined how efficiently they handled time-related text, such as “AM” and “PM” notation. AWS Rekognition and Azure Computer Vision showed promising results for parking sign analysis. Each approach had its strengths and weaknesses. For instance, AWS Rekognition performed significantly better in the recognition of parking sign elements and sub-signs. However, it required an additional algorithmic solution to enhance performance when recognising arrows.

The rest of the paper is organised as follows. Section 2 discusses the related work, both on TSR and PSR methods. Section 3 introduces methodological aspects of our work. Section 4 and Section 5 present the results of our experiments and the corresponding discussion. In Section 6, we analyse the limitations and threats to validity of our study. Finally, Section 7 summarises the paper.

## 2. Related Work

### 2.1. Traffic Sign Recognition

In contrast to the PSR research area, many TSR approaches have been introduced over the last decade. For example, TSR algorithms for intelligent vehicles were proposed in [1,2,3,4,5]. A system for the recognition of traffic signs and traffic lights has been presented in [12]. Another system presented in [13] was trained and evaluated based on the Swedish and German traffic sign datasets [14,15] with the focus on detection of the signs. Some TSR approaches were limited to a particular type of traffic sign. For example, the method proposed in [16] focused on the prohibitory and danger signs, i.e., the signs with a red rim. The method presented in [17] focused on the recognition of warning signs such as “pedestrian crossing” and “children”. A system to detect and recognise text from traffic road signs in various weather conditions was introduced in [18]. An approach for TSR with deep learning has been presented in [19]. The authors aimed to capture and classify traffic signs, focusing on the detection of faded signs and signs obstructed by vegetation.

As this area is rapidly growing, there are also a number of literature reviews (secondary studies) aiming to systematise the research conducted in this field, see Table 1 for an overview. For example, a Systematic Literature Review (SLR) on vision-based autonomous vehicle systems was presented in [20]. Another SLR [6] aimed to address computational methods for TSR in autonomous driving.

Table 2 presents an overview of primary TSR studies, which present either conceptual contributions or corresponding datasets.

It is also important to mention that the acceptance of autonomous vehicles might vary in different countries, influenced by cultural aspects [51]. Therefore, the above-mentioned TSR approaches might also be applied in driver-support systems.

### 2.2. Parking Sign Recognition

An approach for parking sign recognition has been presented as an early prototype in [52]. This approach demonstrated a high accuracy of 96–98%, but the approach was limited to a few particular types of signs, focusing on “no parking” symbol detection and arrow recognition, while the signs with arrows were cropped to allow easier recognition. In our work, we aim to go further and to analyse the AI capacity to identify corresponding elements on the signs that are not cropped and, moreover, might be partially covered by trees or other visual obstacles.

A study focusing on parking sign detection has been presented in [53]. The authors applied a deep learning approach to detect and classify parking signs, without aiming to extract the exact parking constraints from the signs. In contrast to that work, we aim to identify parking constraints from the signs to make sure that a vehicle can be parked in the corresponding slot for the required number of minutes/hours.

An impact of the image resolution on the parking sign recognition and interpretation was investigated in [54]. The study was based on a parking sign dataset collected in Stockholm (Sweden). This work aimed to identify resolution thresholds that maintain acceptable accuracy. Our approach does not cover this aspect.

A study presented in [55] aimed to apply computer vision approaches for automated recognition and localisation of parking signs. The objective of this study was to explore the feasibility of creating a digital map of parking signs. While the research indicated that this direction shows promise, no practical solution has been implemented yet. Given the rapid advancements in AI capabilities, it may be worthwhile to pursue this area further. However, our approach goes in a slightly different direction: we did not aim to create a map of parking signs. Instead, we explore practical solutions for automated recognition of parking signs. For this reason, we investigated the current capacity of AWS Rekognition and Azure Custom Vision for real-time analysis of the parking signs.

## 3. Methods

In this section, we present the background terminology, the methodological aspects of the experiments we conducted, and the methods used for the analysis of the obtained results. We compared the performance of AWS and Azure models on the identification of parking constraints, focusing on the following criteria:

C1: Recognition of parking sign elements (such as clearway, bus zone, no parking, etc.);
C2: Recognition of arrows (denoting constraints applicable to the right, left of or both sides of the parking sign pole) and their directions;
C3: Recognition of sub-signs that are typically used to specify different constraints applicable for different time periods;
C4: Recognition of (in)completeness of parking signs—Check whether a parking sign is complete, i.e., whether the whole sign is presented on the image;
C5: Correct recognition of the text presented on the sign.

As the core performance metrics, we apply precision, recall, and F1 score.

Precision measures the accuracy of positive predictions, i.e., the proportion of correctly identified positives out of all predicted positives:

(1) $$Precision=\frac{TP}{TP+FP}$$

where TP denotes the number of correctly predicted labels (“True Positives”), and FP denotes the number of incorrect predictions (“False Positives”).

Recall measures the model’s ability to identify all actual positives. It shows the proportion of correctly detected positives out of all actual positives:

(2) $$Recall=\frac{TP}{TP+FN}$$

where FN denotes the number of actual labels the model failed to detect (“False Negatives”).

F1 score aims to cover both precision and recall, balancing them. When we need to auto-identify the content of the parking sign, we need both high precision and high recall. Therefore, the F1 score is the most important metric for our experiments.

(3) $$F1=\frac{2\times Precision\times Recall}{Precision+Recall}$$

In the rest of the section, we present the settings of the experiments we conducted to compare the performance of AWS and Azure models.

### 3.1. Criterion C1: Symbol Recognition—Recognition of Relevant Parking Sign Elements

**Figure 1:**
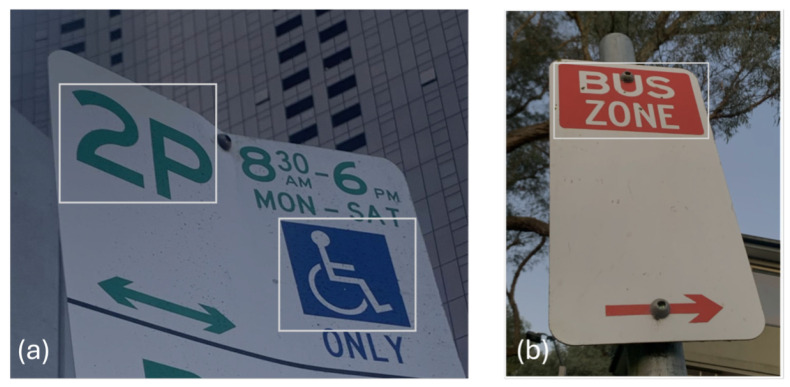
Examples of parking sign symbols highlighted in white: (**a**) a “Park for 2 Hours” and “Disabled” symbol, and (**b**) a “Bus Zone” symbol.

The Symbol Detection model is an AI model within Azure Custom Vision. It is trained to identify symbols on parking signs, see Figure 1. Since we used bounding boxes in the AWS Rekognition Custom Label project, we selected “Object Detection” as the Project Type in this instance. A “bounding box” refers to a rectangular box used in computer vision to define the area of interest in an image. The bounding box is defined by the following parameters:

- Coordinates: The top-left corner (often represented as x, y coordinates) and the width and height of the box.
- Width and Height: The dimensions of the rectangle that encloses the object.

For example, in the image presented in Figure 1b, the bounding box (the white rectangle) highlights the “bus zone” area.

This choice enables us to detect specific objects within an image, mirroring the functionality of bounding boxes in the AWS Rekognition project. We opted for the General domain, as it offers the best balance of cost and suitability for our project’s requirements. Table 3 presents a summary of testing data. The slight difference in the size of the training datasets between AWS and Azure arose from Azure’s requirement of “at least 15 training images for each label”. To meet this requirement, we slightly modified some of the existing training images for labels with fewer than 15 samples. Specifically, we cropped these images slightly (by 0.1 cm) to effectively increase the number of training samples. Consequently, the training dataset for Azure Custom Vision contained slightly more images than the AWS Rekognition Custom Labels dataset. Since the augmentation involved reusing existing images with minor modifications, it should not impact the model’s results.

To create the dataset for this experiment, we followed two methods: (1) The authors captured photos of the parking signs using smartphone cameras in high-efficiency mode and a standard resolution of 24 MP. In the case of a complex sign, we cropped the image to include only one sub-section of a parking sign. (2) We also utilised digital parking signs provided by Manningham City Council [56]. As the digital images obtained from [56] are generally clearer than images obtained by photos (no obstruction by vegetation, perfect light conditions and frontal view, etc.), we aimed to have no more than 30% of the images of this kind in our training and testing data set for this experiment.

To ensure consistency between the AWS and Azure models, the same threshold level for each label used in AWS was applied when testing the Azure models. A threshold refers to the confidence score required for the model to classify a prediction as belonging to a specific label. For example, if the threshold is set to 80%, the model must be at least 80% confident in its prediction for the label to be considered correct. Table 4 summarises the information on the labels, the corresponding number of images used for training and testing, and the threshold used for testing each label. In AWS models, the “assumed threshold” for each label is automatically calculated by AWS Rekognition Custom Labels. This threshold refers to the confidence percentage for each predicted label. The assumed threshold cannot be manually set; it is calculated based on the best F1 score achieved on the test dataset during model training.

To automate the process of uploading images, drawing bounding boxes around parking symbols, and testing over 1000 images, three Python 3.9 scripts were used:

- aws_to_azure_bbox_converter.py: This script was used to convert a training manifest file from AWS (training_aws_annotations.json) into a format compatible with Azure (training_azure_annotations.json). The AWS manifest, exported from an AWS model, contains detailed information about the training images, including image names, paths to AWS S3 storage, bounding box coordinates, and labels for each bounding box.
- azure_custom_vision_uploader.py: This script utilised the Azure-compatible training manifest to interact with the Azure Custom Vision API. It automated the process of uploading images and adding bounding boxes with corresponding labels to each image.
- test-bulk.py: This script was used to run tests on the images and automatically draw bounding boxes around the parking symbols. It simplified the process of quickly reviewing the results and evaluating the performance of the model.

To train the model, we utilised the “Advanced Training” option within the constraints of the free tier. We limited the training duration to a maximum of 1 h to optimise model accuracy, while ensuring that we stayed within the usage limits of the free tier.

### 3.2. Criterion C2: Recognition of Arrows

**Figure 2:**
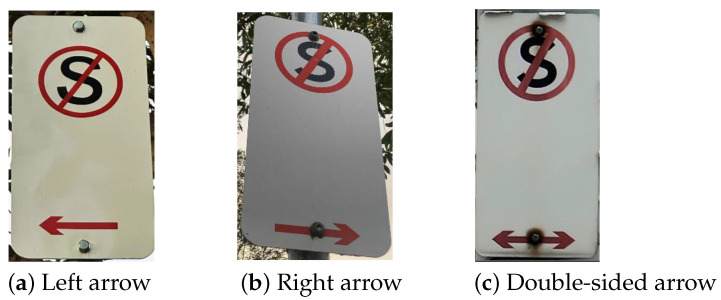
The range of arrow types found on parking signs in Melbourne.

**Figure 3:**
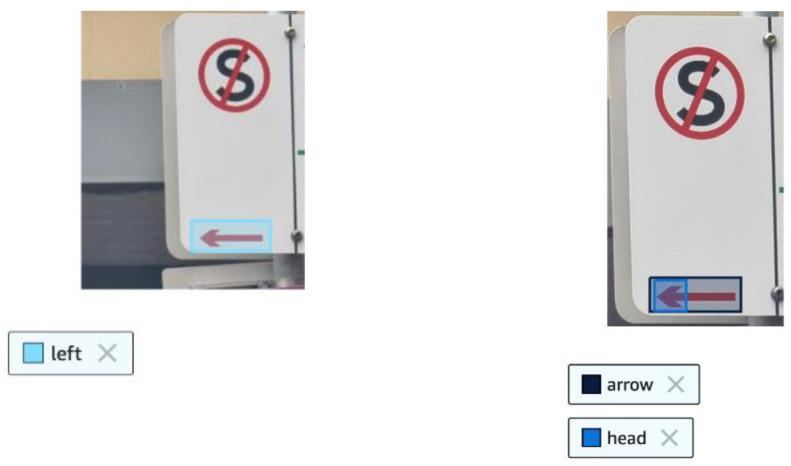
Different approaches used to determine the arrow direction.

This test aims to compare the performance of image object classification models in AWS and Azure to detect the direction of arrows in parking signs. As shown in Figure 2 below, parking signs in Melbourne can contain right, left and double-sided arrows. To analyse the model’s capacity to detect arrows correctly, the following approaches can be used:

(1) A model can be trained in AWS or Azure by selecting the arrow by a bounding box and labelling it as “left”, “right”, or “both-sides”; see Figure 3a. The trained model can then be called using new images, and the model will detect the desired object (the arrow) and predict a label (left, right or both-sides).
(2) Another approach would be to use separate labelling, see Figure 3b, where two bounding boxes are used: one around the entire arrow, and another around the arrow head. Then, based on the position location of the bounding boxes, the arrow direction can be calculated using an algorithmic solution.

In our experiments to compare the AWS vs. Azure performance, we apply the first approach. This allows us to directly compare the ability of the model to detect arrows, rather than comparing the workaround method that relies on algorithmic calculations. As the dataset, we applied the same 64 training and 48 testing images that were used for the experiments presented in Section 3.1. The dataset covered all three arrow types—left, right and both sides, see Table 5. To create the dataset, the photos of the parking signs were taken by the authors using smartphone cameras in high-efficiency mode and a standard resolution of 24 MP. To analyse cases with a single arrow on a sign, the photos were cropped correspondingly, as can be seen in an example in Figure 3.

All resources utilised in this project are within the free tier. Since we used bounding boxes in the AWS Rekognition Custom Label experiments, we selected “Object Detection” as the Project Type in this instance. This choice enables us to detect specific objects within an image, mirroring the functionality of bounding boxes in the AWS Rekognition project. We opted for the General domain, as it offers the best balance of cost and suitability for our project’s requirements. As explained in Section 6, AWS allows us to provide a testing dataset, which it uses to calculate performance scores based on automatically calculated thresholds, which were 0.633 for both-side arrows, 0.521 for left arrows, and 0.540 for right arrows. To compare the performance with Azure, we have used the same testing dataset, manually called the Azure model API setting the threshold ourselves, checking the predicted labels and manually calculating the precision, recall and F1 scores. In order to have similar settings in Azure, we ran the Azure model using the AWS-calculated thresholds for each label.

### 3.3. Criterion C3: Recognition of Sub-Signs

**Figure 4:**
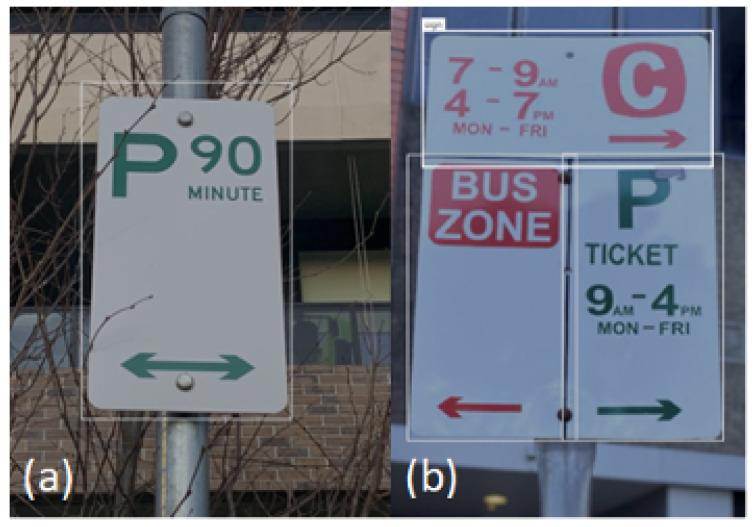
Examples of a sub-sign detection: (**a**) a simple parking sign (consisting of a single “sub-sign”), (**b**) a complex parking sign with multiple “sub-signs”.

We aimed to detect the presence of sub-signs within a parking sign. We use the label “sign” to denote the presence of a sub-sign. A simple sign would consist of a single sub-sign, as presented in Figure 4a. A complex parking sign would have multiple sub-signs. For example, the sign presented in Figure 4b has three sub-signs.

In our experiments, we used a dataset of 178 images (145 images were used for training and 33 images were used for testing). To create the dataset for this experiment, the authors captured photos of the parking signs using smartphone cameras in high-efficiency mode and a standard resolution of 24MP. Since we used bounding boxes in the AWS Rekognition Custom Label project, we selected “Object Detection” as the Project Type in this instance. This choice enables us to detect specific objects within an image, mirroring the functionality of bounding boxes in the AWS Rekognition project.

### 3.4. Criterion C4: Recognition of (In)Completeness of Parking Signs

**Figure 5:**
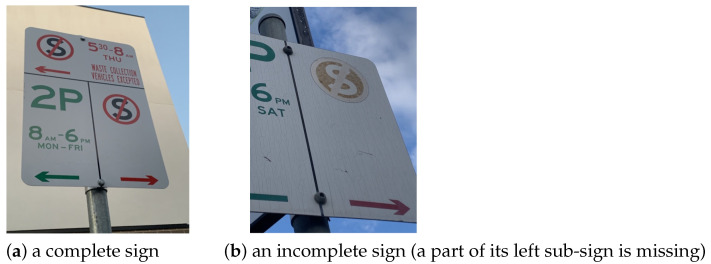
Examples of complete and incomplete photos of the parking signs.

Another important parameter to check is whether a model can correctly identify the completeness of the sign, i.e., whether some parts of the sign are missing on the photo; see Figure 5. The Azure model uses the image classification approach, which is different from the previous models where a bounding box was used. Image classification applies one or more labels to the whole image instead of parts of an image, as per object detection. This is equivalent to image-level labels in AWS Rekognition Custom Labels. Thus, we applied two labels here, “complete” and “incomplete”. An image is considered “complete” when it contains the entire parking sign (all four edges and corners are visible). Correspondingly, an image is considered “incomplete” when part of the parking sign is cut out of the image (not all four edges and corners are visible in the image).

As the dataset, we used 441 images (352 images have been used for training and 89 images have been used for testing), where 173 of the images presented incomplete signs and 179 images presented complete signs, see Table 6. To create the dataset for this experiment, the photos of the parking signs were taken by the authors using smartphone cameras in high-efficiency mode and a standard resolution of 24 MP. The images of incomplete signs were created by two methods: (1) an original photo contained only a part of the sign, and (2) the original photo was cropped to include only a part of a parking sign.

### 3.5. Criterion C5: Text Recognition

Another important parameter to analyse is the capabilities of AWS Rekognition and Azure Computer Vision to accurately capture the text presented on the parking signs. Both platforms offer a managed service to extract text from images. In our experiments, we have not conducted any training of the models to analyse this parameter. The data set used for this experiment included 51 images. To create the dataset for this experiment, the photos of the parking signs were taken by the authors using smartphone cameras in high-efficiency mode and a standard resolution of 24MP. We cropped to include only one sub-section of a parking sign. We focused on the following sub-criteria: extra text detection, missing text, and order of the text. In what follows, we discuss them in more detail.

(C5.1) No extra text detected. This criterion is considered passed if the system detects only the relevant text in the parking sign without including any irrelevant text. If irrelevant text or incorrect time markers are detected, it could be passed to the LLM or further analysis. This can alter or distort the time information on the parking sign, leading to incorrect interpretations and potentially miscalculating the parking verdict.

(C5.2) Correct order of the text. This criterion is considered passed for parking signs if the time frames, including AM/PM markers, are detected exactly as they appear on the parking sign. If the text order is incorrect (e.g., detecting “7 PM to 4 AM” instead of “7 AM to 4 PM”), it can completely change the meaning of the parking sign. This incorrect interpretation will affect the parking verdict calculation and result in a wrong parking decision.

(C5.3) No text missed. This criterion is considered passed if no essential text is missing from the sign, such as time markers or day ranges. If important elements like AM/PM, specific days (e.g., Mon-Sat), or time frames are missing, it can lead to incomplete or incorrect parking verdicts. The lack of crucial markers results in losing essential information needed to accurately calculate the parking restrictions.

Experiments with AWS Rekognition: The OCR process was driven by the DetectText API, which scans images and extracts any text it finds. The parking sign images were stored in an S3 bucket, and an AWS Lambda function was set up to automatically handle the text extraction. Every time a new image was uploaded to the S3 bucket, the Lambda function would automatically trigger the DetectText API, which would then extract the text from the image. This setup allows for a fully automated OCR process, meaning no manual effort is needed once it is configured. The DetectText API provides a structured response with the extracted text, which can then be used for further analysis. To compare the results, we used this method to validate the setup, ensuring that the OCR process was extracting text accurately.

Experiments with Azure: Vision Studio, an interactive tool within Azure, was used for testing the OCR functionality. This tool provides a user-friendly interface for uploading images and processing them to extract text. Alternatively, to perform OCR using Azure’s Computer Vision API, a cURL command can be used to send an image for text extraction. After sending the request, the API returns a JSON response containing the extracted text and bounding box coordinates around the text. To make sure the results were consistent and reliable, we cross-validated the OCR outputs from both Vision Studio and the API method (cURL). Since both methods use the same underlying API, we expected the results to align. This helped confirm that the OCR process was working correctly and that the extracted text was accurate, regardless of the method used.

## 4. Results

In this section, we present the results of our experiments on the detection of parking symbols, arrows, and sub-signs, as well as on the completeness checks and text recognition.

### 4.1. Criterion C1: Symbol Recognition

The Azure Custom Vision model achieved an F1-score of 0.848, while the AWS Rekognition Custom Labels model showed a significantly higher F1-score of 0.991; see Table 7. Based on these results, we can conclude that, under the same training technique, training images, and testing threshold, the AWS Rekognition model outperforms Azure Custom Vision in detecting parking symbols.

### 4.2. Criterion C2: Recognition of Arrows

As shown in Table 8, the Azure model significantly outperforms the AWS-equivalent model (F1 score of 0.9 compared to 0.4). Table 9 presents a more detailed comparison, where an arrow type is also taken into account. This allows us to conclude that the core difference in the performance of the models is in the ability to detect left and right arrows. Both AWS and Azure models have good performance in detecting both-sided arrows, with very similar values of F1 scores (0.944 and 0.950, respectively). However, the performance for the left and right arrows in AWS is much lower.

### 4.3. Criterion C3: Recognition of Sub-Signs

Table 10 presents a summary of the performance comparison for the sub-sign detection. Overall, the AWS model identified sub-signs with a significantly higher accuracy. It has perfect precision, recall and thus an F1-Score of 1.000 with a very high confidence level of 94%. Thus, each sub-sign in the testing images was correctly identified with no false positives (where part of the image that is not a sub-sign is flagged with a “sign” label) or false negatives (where a valid sub-sign was not flagged with a “sign” label). For the same confidence level of 94%, Azure’s model is robust against false positives with 100% precision but performs poorly at preventing false negatives with just 35.4% recall. This lowers the F1-Score to 52.3%.

### 4.4. Criterion C4: Recognition of (In)Completeness of Parking Sign Images

Both models performed on a similar level of accuracy. The AWS model achieved an F1 score of 0.933, while the Azure model achieved a higher F1 score of 0.976, see Table 11. AWS incorrectly labelled six parking signs as “incomplete”, whereas Azure incorrectly labelled four parking signs as “incomplete”. Neither AWS nor Azure models labelled any incomplete parking sign as “complete”.

### 4.5. Criterion C5: Text Recognition

**Figure 6:**
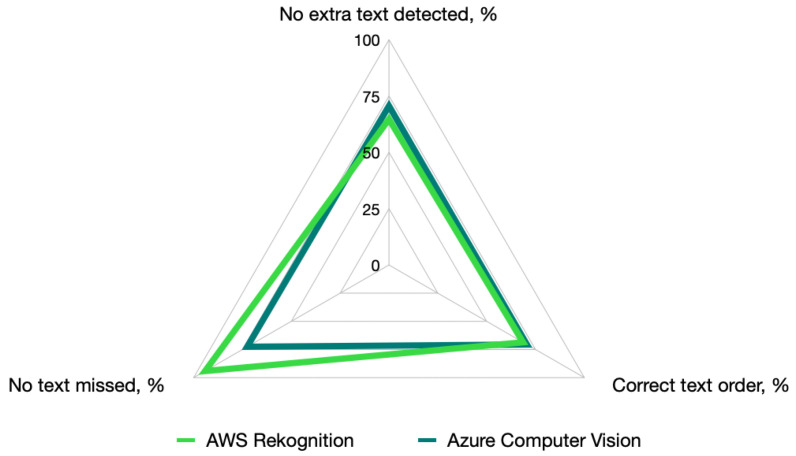
Comparison of the performance of the AWS and Azure models in text recognition on the parking signs.

Figure 6 presents a summary of the performance comparison in the following three dimensions of text recognition

Criterion C5.1: No extra text detected. AWS Rekognition detected extra text on 18 parking sign images, which included redundant time markers (such as “PM”) and irrelevant symbols. For example, AWS incorrectly identified an extra “S” in “BUS ZONE” and also detected symbols like the no-standing symbol as the letter “S”. Azure Computer Vision detected extra text on 15 parking sign images, particularly redundant “AM” or “PM” markers. However, Azure performed better than AWS in avoiding irrelevant symbols, making it slightly more accurate in this area.

Criterion C5.2: Correct order of the text. AWS Rekognition model: Incorrect text order was observed in 16 parking sign images. For example, in time-related text like “7 AM–10 PM”, AWS sometimes swapped the markers, resulting in “7 AM PM 10”. Azure Computer Vision: Incorrect text order was observed in 15 images. Similar to AWS, the time markers (“AM” and “PM”) were sometimes out of place.

Criterion C5.3: No text missing. AWS Rekognition missed text in three images of parking signs, specifically time markers such as “PM”. For example, in the case of the time range “10 PM”, AWS detected only “10” while failing to detect the “PM”. Azure Computer Vision missed text in 14 images of parking signs, also typically related to the time markers. For example, in the case of the time range “7 AM–4 PM”, Azure only detected “7 to 4” without specifying “AM” or “PM”, leading to a loss of key contextual information.

## 5. Discussion

**Figure 7:**
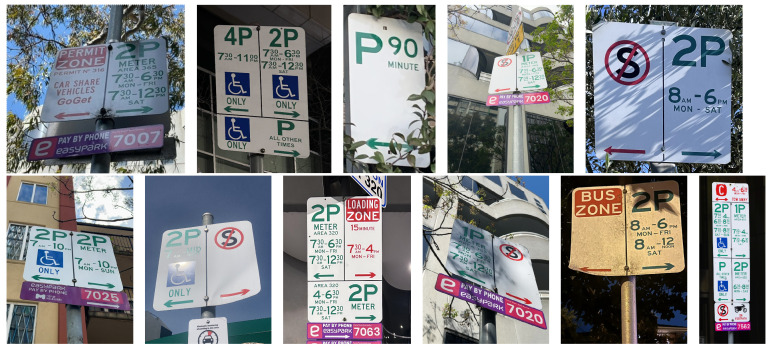
Examples of photos of parking signs made in different conditions.

Our work is the first to employ AI-based approaches for (1) recognising parking signs that are typical for Melbourne, Australia, (2) extracting parking constraint information from them. Thus, our analysis was based on a new proposed dataset that has not been used in any prior studies. The dataset included photos of parking signs made in different weather/light conditions as well as from different distances from the signs; see Figure 7. We aimed to cover not only different types of parking constraints but also different complexities of signs. Using the new proposed dataset also means that we cannot directly compare the results of our study with the results of related works. Generally, there are many approaches to traffic sign detection and recognition, but the number of approaches focusing on parking sign recognition is very limited. One of the reasons for this might be significant variations in parking regulations and parking sign formats among the countries and cities. Table 12 presents a summary comparison of the proposed approach to the related PSR studies.

**Figure 8:**
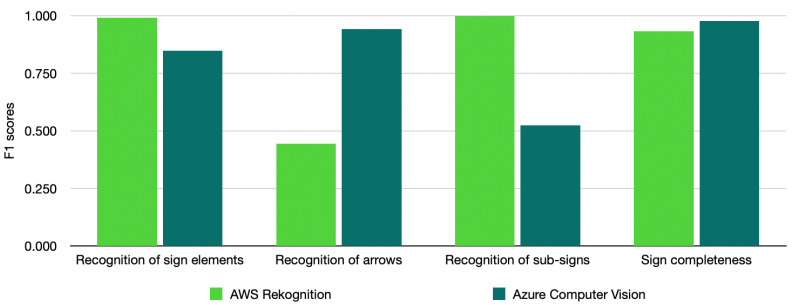
Overview of the performance comparison of the AWS Rekognition and Azure Computer Vision models.

Our study is also the first to compare the performance of AWS Rekognition and Azure Computer Vision models in the context of parking sign recognition. To summarise the results of our experiments, Figure 8 presents an overview of the performance comparison of the AWS and Azure models based on their F1 scores.

AWS Rekognition significantly outperforms Azure Computer Vision in the following criteria, which makes it a more promising option for further analysis and enhancements:

C1:   Recognition of parking sign elements;
C3:   Recognition of sub-signs;
C5.3:  Correct recognition of the text. No text missed.

Azure Computer Vision performs significantly better in the criterion C2 (Recognition of arrows) if an approach with direct labelling is used. However, this can be compensated if we apply an approach that applies a separate labelling of arrows and arrow headers with a corresponding algorithmic solution to calculate the type of arrows (left, right, or double-sided).

AWS Rekognition and Azure Computer Vision have a similar level of performance, with Azure Computer Vision providing slightly better results, in the following criteria:

C4:   Recognition of (in)completeness of parking signs;
C5.1:  Correct recognition of the text. No extra text detected;
C5.2:  Correct recognition of the text. Correct order of the text.

Neither AWS Rekognition nor Azure Computer Vision has provided F1 score values of 1.000 for all criteria, which means they cannot be currently applied directly for parking sign analysis. However, further training of the models on a larger dataset might provide better results. Moreover, an algorithmic fine-tuning can also be helpful to deal with C2 and C5.

## 6. Limitations and Threats to Validity

There are several limitations and threats to the validity of our experiments. This section summarises several threats that may have occurred in our research and their respective mitigation strategies.

External threats to validity refer to the transferability of obtained results: The first threat is the limited scope of the dataset used for our experiments, which was limited to 1225 images of parking signs. To make the dataset representative, we aimed to cover the typical types of signs we observe in Melbourne. We used photos taken from different distances and angles as well as in different light conditions and under different impacts from shadows and obstructions. However, our set is definitely not covering all possible variants of parking signs. It might be useful to expand the dataset with images taken under a broader range of weather conditions, such as heavy fog, thunderstorms, etc.

Both AWS and Azure models are cloud-hosted, which means they depend on having a stable Internet connection. As our study is especially relevant for parking in large cities, we consider the assumption regarding Internet connection as reasonable. In remote areas, where the connection might be unstable or unavailable, the parking signs are typically much simpler, containing only a single subsection. The benefit of using an automated PSR system would be limited in these conditions.

Internal threats to validity refer to the potential/unexpected factors that might influence the outcome of the study. Our research method in Section 3 involves manual steps to calculate the performance statistics (precision, recall and F1 score). While these statistics are automatically calculated for the AWS models, we had to calculate them manually for the results produced by Azure models. After the calculations have been done by an author working on the corresponding set of experiments, the results have been reviewed by another author to avoid typos or miscalculations.

Also, in AWS models, the “assumed threshold” for each label is automatically calculated by AWS Rekognition Custom Labels. This threshold refers to the confidence percentage for each predicted label. The assumed threshold cannot be set manually, as it is calculated based on the best F1 score achieved on the test dataset during model training. To achieve a similar comparison in Azure, we called the Azure model API using the same test images used for AWS and manually calculated the F1 score. When calling the model API, we manually set the confidence threshold to be the same as AWS’ threshold for each label. However, it should be noted that these thresholds could potentially favour the AWS model as it is calculated based on the best F1 score achieved for the AWS model during testing.

## 7. Conclusions

In this paper, we presented the key findings of a research project designed to address the research question: (RQ1) How can parking signs be analysed using AWS Rekognition and Azure Custom Vision?

Our study offers the following novel contributions: (1) This is the first comparison of the performance of AWS Rekognition and Azure Computer Vision models specifically for parking sign recognition. (2) This is the first AI-based approach to recognising parking signs typical for Melbourne, Australia, and extracting parking constraint information from them.

We utilised 1225 images (created under different weather/light conditions, etc.) of the parking signs to evaluate the capabilities of AWS Rekognition and Azure Custom Vision models based on the following criteria:

C1: Recognition of parking sign elements;
C2: Recognition of arrows;
C3: Recognition of sub-signs;
C4: Recognition of (in)completeness of parking signs;
C5: Correct recognition of the text presented on the sign.

AWS Rekognition demonstrated better results within C1 and C3 criteria (F1 scores of 0.991 and 1.000 by AWS vs. 0.848 and 0.523 by Azure, respectively), as well as one sub-criterion of C5 (C5.3, “No text missed”, 0.94 by AWS vs. 0.73 by Azure model). While Azure Computer Vision performed better in C2 (F1 score only 0.444 by AWS vs. 0.941 by Azure model), this advantage could be offset by utilising an algorithmic approach to achieve an F1 score of 1.000. For other criteria, both approaches demonstrated a similar level of performance: For C4, F1 scores were 0.933 by AWS and 0.976 by the Azure model. For C5.1 and C5.2, we obtained 0.65 and 0.69 by AWS and 0.711 and 0.71 by Azure model, respectively. While neither of them provided F1 score values of 1.000 for all criteria, this study demonstrated that the current capacity of both AWS Rekognition and Azure Custom Vision is close to being ready for live application on parking sign analysis. We consider the results demonstrated AWS Rekognition models as more promising for further refinements to achieve 100% accuracy of automated analysis of parking signs.

Future Work: As a promising future work direction, we consider further training of the models on a larger dataset to attempt obtaining improved results. Additionally, fine-tuning the algorithms may help address the challenges associated with criteria C2 and C5. It would make sense to analyse whether there is some correlation between the type of parking sign and the AI models, and adjust the training data sets accordingly.

Another promising research direction would be to investigate the capacity of deep learning approaches that can be used in offline settings. Automated PSR systems would provide the most benefit in large cities, where parking signs are complex, but the Internet connection allows utilising AWS and Azure services. Nevertheless, an offline solution might provide additional benefits towards fully automated driving.

## Figures and Tables

**Table 1:** Literature reviews on TSR-related studies.

Reference	Review Type	Years Covered	Focus
[6]	SLR	2000–2024	recognition methods
[20]	SLR	2011–2021	DL approaches
[9]	review	2017–2022	ML and DL approaches
[21]	review	2010–2020	benchmark datasets, detection methods
[22]	review	2000–2018	benchmark datasets, detection methods
[23]	review	1999–2016	detection methods

**Table 2:** Related primary studies on TSR methods.

Ref.	Type	Focus
[9]	dataset	overview of the traffic sign datasets
[10]	dataset	dataset of traffic signs from Germany
[15]	dataset	dataset of traffic signs from Germany
[11]	dataset	datasets of traffic signs from Belgium and the Netherlands
[14]	dataset	datasets of traffic signs from Sweden
[1]	conceptual	TSR algorithm for intelligent vehicles
[2]	conceptual	TSR algorithm for intelligent vehicles
[3]	conceptual	TSR algorithm for intelligent vehicles
[4]	conceptual	TSR algorithm for intelligent vehicles
[5]	conceptual	TSR algorithm for intelligent vehicles
[24]	conceptual	TSR algorithm for intelligent vehicles
[12]	conceptual	system for recognition of traffic signs and traffic lights
[13]	conceptual	system for sign detection, trained and evaluated based on the Swedish and German traffic sign datasets
[25]	conceptual	approach to detect traffic signs based on such properties as colour and shape
[26]	conceptual	enhanced algorithm for traffic sign detection
[27]	conceptual	framework for detecting and reconstructing traffic sign images
[12]	conceptual	system for traffic sign/light recognition
[17]	conceptual	method for recognition of warning signs
[18]	conceptual	system to detect and recognise text from traffic signs in various weather conditions
[16]	conceptual	method to recognise the prohibitory and danger signs
[19]	conceptual	DL-based approach to capture and classify traffic signs, focusing on the detection of faded signs and signs obstructed by vegetation
[28]	conceptual	DL-based approach for traffic-sign detection and recognition
[29]	conceptual	DL-based approach for traffic sign detection
[30]	conceptual	DL-based approach for traffic sign detection
[31]	conceptual	DL-based approach for traffic sign detection
[32]	conceptual	DL-based approach for detection of Vietnamese traffic danger and warning signs
[33]	conceptual	transfer learning method for the recognition of Pakistani traffic signs
[34]	conceptual	TSR method for in-car cameras
[35]	conceptual	TSR method based on monocular camera
[36]	conceptual	TSR model to enhance real-time detection efficiency
[37]	conceptual	TSR method focusing on the issues related to scale and rotation
[38]	conceptual	TSR method focusing on snow conditions
[39]	conceptual	TSR method focusing on complex weather conditions
[40]	conceptual	TSR method based on semantic scene understanding and structural traffic sign location
[41]	conceptual	framework for detection performance of small traffic signs in complex backgrounds
[42]	conceptual	TSR method focusing on Indian traffic signs
[43]	conceptual	TSR method focusing on Indian traffic signs
[44]	conceptual	TSR method focusing on Indian traffic signs
[45]	conceptual	TSR method focusing on Mexican traffic signs
[46]	conceptual	TSR method aiming to cover worldwide cross-regional traffic signs
[47]	conceptual	real-time TSR method
[48]	conceptual	real-time TSR method
[49]	conceptual	real-time TSR method
[50]	conceptual	TSR method focusing on small signs

**Table 3:** Symbol detection: dataset sizes.

	Azure Custom Vision	AWS Rekognition Custom Labels
Training dataset	979	960
Testing dataset	265	265

**Table 4:** Image counts and threshold values per label. (*) denotes that the augmentation technique was applied to these labels in Azure Custom Vision model to meet Azure’s requirement of having “at least 15 images” per label.

Labels	Nr. of Training Images	Nr. of Testing Images	Threshold
bus	20	5	0.841
clearway	48	20	0.929
disabled	36	9	0.850
park-unlimited	48	13	0.920
park-2 h	166	40	0.490
park-3 h	29	8	0.904
park-4 h	41	11	0.820
loading-zone	31	8	0.890
mail-zone (*)	8	3	0.853
no-parking	34	10	0.925
no-standing	214	57	0.882
park-10 min	15	5	0.804
park-15 min	38	14	0.811
park-1 h	84	22	0.938
park-2 min (*)	9	4	0.440
park-30 min	40	10	0.875
park-5 min	17	5	0.452
park-90 min (*)	14	3	0.106
permit	43	11	0.932
taxi (*)	14	4	0.685
works-zone (*)	11	3	0.723

**Table 5:** Arrow recognition: sizes of datasets used for training and testing.

	Training Dataset	Testing Dataset
Left	30	16
Right	17	13
Both-sides	17	19
Total	64	48

**Table 6:** Completeness identification: sizes of datasets used for training and testing.

	Training Dataset	Testing Dataset
Complete parking sign	179	44
Incomplete parking sign	173	45
Total	352	89

**Table 7:** Comparison of the performance of the AWS and Azure models used to detect parking symbols.

	Precision	Recall	F1
AWS Rekognition Custom Labels	0.988	0.996	0.991
Azure Custom Vision	0.950	0.790	0.848

**Table 8:** Comparison of the performance of the AWS and Azure models used to detect arrow direction. As a threshold, the AWS- calculated thresholds have been applied.

	Precision	Recall	F1
AWS model	0.478	0.443	0.444
Azure model	0.906	0.980	0.941

**Table 9:** Detailed comparison of the performance of the AWS and Azure models used to detect arrow direction, broken down by custom label.

Model	Label	Precision	Recall	F1
AWS model	left	0.154	0.250	0.190
	right	0.333	0.140	0.197
	both-sides	0.947	0.940	0.944
Azure model	left	0.842	1.000	0.914
	right	1.000	0.929	0.963
	both-sides	0.905	1.000	0.950

**Table 10:** Comparison of the performance of the AWS and Azure models used to identify sub-signs with the confidence level threshold of 0.94.

	Precision	Recall	F1
AWS model	1.000	1.000	1.000
Azure model	1.000	0.354	0.523

**Table 11:** Comparison of the performance of the AWS and Azure models used to detect (in)completeness of parking sign images.

	Precision	Recall	F1
AWS model	0.867	1.000	0.933
Azure model	0.911	1.000	0.976

**Table 12:** Comparison of the proposed approach to the related PSR studies.

Reference	Focus	Type of Parking Signs	Parking Constraints
[55]	digital map of parking signs	all signs	not covered
[52]	sign recognition	“no parking”, arrow	not covered
[53]	sign detection and classification	all parking signs	not covered
[54]	resolution threshold analysis	all parking signs	not covered
Proposed solution	sign recognition	all parking signs	covered

## Data Availability

The data presented in this study are available on request from the corresponding author.

## Abbreviations

The following abbreviations are used in this manuscript:

AI	Artificial Intelligence
AWS	Amazon Web Services
DL	Deep Learning
ML	Machine Learning
PSR	Parking Sign Recognition
SLR	Systematic Literature Review
TSR	Traffic Sign Recognition
